# The Expression of Notch/Notch Ligand, IL-35, IL-17, and Th17/Treg in Preeclampsia

**DOI:** 10.1155/2015/316182

**Published:** 2015-05-14

**Authors:** Weiping Cao, Xinzhi Wang, Tinmei Chen, Huaying Zhu, Wenlin Xu, Songlan Zhao, Xiaoqing Cheng, Liangping Xia

**Affiliations:** ^1^Department of Obstetrics, Maternity and Child Health Hospital of Zhenjiang, No. 20 Zhengdong Road, Zhenjiang, Jiangsu 212001, China; ^2^Jiangsu Key Laboratory of Drug Screening, China Pharmaceutical University, 24 Tong Jia Xiang, Nanjing 210009, China; ^3^Central Laboratory of Medicine, Maternity and Child Health Hospital of Zhenjiang, No. 20 Zhengdong Road, Zhenjiang, Jiangsu 212001, China; ^4^Obstetrics and Gynecology, The Affiliated People Hospital of Jiangsu University, Zhenjiang, Jiangsu 212001, China; ^5^Laboratory of Medicine, Maternity and Child Health Hospital of Zhenjiang, No. 20 Zhengdong Road, Zhenjiang, Jiangsu 212001, China

## Abstract

The aim of this study was to examine the interaction of Notch/Notch ligand with Th17/Treg, cytokines IL-35 and IL-17 in cases of preeclampsia (PE). *Methods.* Peripheral blood was obtained from 42 PE patients and 22 health pregnant women. The mRNA expressions of Notch/Notch ligand, Treg transcription factor FoxP3 and Th17 transcription factor ROR*γ*t, EBI3 and P35 (IL-35 two subunits), and IL-17 were determined by qPCR. The serum levels of IL-17 and IL-35 were measured by ELISA. *Results.* It was observed that the expressions of Foxp3, EBI3, and P35 in PE patients were lower compared with normal pregnancy, whereas the ROR*γ*t expression was significantly increased. The results also demonstrated that PE patients exhibited decreased levels of Treg-related cytokine IL-35, whereas IL-17 was significantly increased. PE patients expressed higher levels of Notch receptor (1–4) and Notch ligand of DLL4, whereas Notch ligand of Jagged-1, -2 was much lower. Furthermore, the levels of FoxP3 T cells correlated positively with Jagged-2. In addition, there were positive correlations between the mRNA level of IL-17 and DLL4. *Conclusion.* Our results indicated that maternal immunological changes may reverse maternal tolerance in PE, and this phenomenon may due to the Th17/Treg imbalance affected by Notch/Notch ligand.

## 1. Introduction

Preeclampsia (PE) is a major cause of maternal and neonatal mortality that occurs in 3%–10% of all pregnancies in China [1]. PE is characterized by the development of maternal high blood pressure (>140/90 mmHg) and proteinuria (>300 mg/24 h) in the second half of pregnancy [2]. The etiology of PE is not fully understood. Although PE may occur due to angiogenic imbalance, chronic inflammation, inadequate tolerance, and hypoxia, in certain cases no cause is identified. Pregnancy can be considered as a physiological miracle in which an event that is normally forbidden, propagation of foreign tissue, is accommodated for a defined period of time by the immune system. Given our observation of the clinical disease, an immune mechanism is plausible. Over the past few decades, extensive research has provided data to support the hypothesis that PE has also been linked to immune intolerance [3, 4]. More recently, a role for regulatory T cells (Tregs) in the development of PE has been proposed. Tregs are regarded as having an important role in regulating the immune response and tolerance to the fetus. Treg cells are a subpopulation of CD4^+^CD25^+^ lymphocytes specifically characterized by the specific transcription factor Forkhead Box protein 3 (FoxP3) [5–7]. It has been shown that Treg cell function requires the expression of Foxp3. Since first designated in 2007, IL-35 has drawn wide attention as an important immunosuppressive cytokine [8]. IL-35 is secreted from regulatory T cells and is an anti-inflammatory cytokine suppressing the immune response through the expansion of Tregs and suppression of Th17 cells development [9, 10]. IL-35 is composed of a P35 and EBI-3 subunit. Recently, a new T cell subset was recognized as a key effector T cell. These Th17 cells, which secrete IL-17, are thought to play a role in chronic inflammation and protection from fungal infection [11]. Th17 cells produce proinflammatory cytokines, such as IL-17 and IL-22. The IL-17 family contains six members (IL-17A–F), with IL-17A being designated as the prototypic of IL-17 cytokine [12]. The transcriptional factor to develop Th17 cells is acid-related orphan receptor *γ*t (ROR*γ*t) in humans. Numerous studies of recurrent pregnancy loss, reproductive failure, and pregnant failure in humans have revealed a close association of these conditions with the increased production of Th17 cells [13–15]. An elaborate immune balance between immune effectors and immune regulators is crucial to achieve, implant, and maintain pregnancy until term. The purpose of this study was to investigate whether the Treg/Th17 balance was broken in patients with PE.

The Notch signaling system is conserved from* Drosophila* to humans and regulates cell differentiation, proliferation, and survival. In mammals there are four Notch receptors (1–4) and five Notch ligands (Jagged-1, Jagged-2, Delta-like 1 (Dll-1), Dll-3, and Dll-4) [16]. Notch plays multiple roles in T cell development in the thymus and T cell differentiation in the periphery [17, 18]. The second purpose of this study was to investigate the expression of Notch/Notch ligands in peripheral blood of PE patients, because Notch signaling plays a critical role in proliferation and differentiation of human first-trimester T helper cells.

The pathogenesis of PE is complicated and multiple factors are involved in the formation of a clear clinical picture. We propose the hypothesis that preeclampsia is associated with the imbalance of Th17/Treg cells, and for this propose we measured the concentrations of IL-17 and IL-35 in the serum and detected the mRNA expression levels of IL-17 and IL-35 subunits (P35 and EBI3), ROR*γ*t, and Foxp3 in the periphery PBMC cells of preeclampsia and normal pregnant women. We propose that Notch affects Th17/Treg imbalance, which may be responsible for the pathogenic mechanism of development and progression of PE. To date, there have been no data regarding IL-17 and IL-35, ROR*γ*, FoxP3, and Notch/Notch ligand on the immune system with PE patients.

## 2. Materials and Methods

### 2.1. Study Protocol

The protocol was in conformity with the ethical guidelines of our institution and this study has the approval of the Ethics Committees of the Maternity and Child Health Hospital (Zhenjiang, China). Written consent was obtained from all subjects following a full explanation of the procedure.

### 2.2. Study Population

The study included 22 healthy pregnant women and 42 pregnant women suffering from characteristic gestation-associated diseases mild preeclampsia (*n* = 22) and severe preeclampsia (*n* = 20). Preeclampsia patients were from the Department of Obstetrics of Zhenjiang Maternity and Child Health Care Hospital (Zhenjiang, Jiangsu Province, China), who received treatment between June 2013 to October 2014. Preeclampsia was diagnosed as blood pressure higher than 140/90 mmHg at two separate occasions, 6 h apart, along with significant proteinuria (>300 mg protein in a 24-hour collection or a dipstick reading of >2+ on a voided random urine sample in the absence of urinary tract infection) in previously normotensive women. Preeclampsia was considered severe if the systemic blood pressure was greater than 160/110 mmHg or if there was consistent proteinuria of more than 5 g/day. In addition, any patient with cerebral or visual disturbances, epigastric pain, pulmonary edema, oliguria (400 mL, or less in 24 hours), or an abnormal platelet count and liver function profile was included in the severe preeclampsia group (including HELLP syndrome) [1]. Women with other disorders of the immune system or using (other) immune suppressing medication were excluded from this study. All clinical characteristics of preeclampsia and normal control are shown in Table 1.

### 2.3. Blood Sample Preparation

Venous blood ~5 mL was obtained by venipuncture from mild and severe PE, and healthy pregnant women. Of the 5 mL, 3 mL was heparinized for the isolation of peripheral blood mononuclear cells (PBMCs), while the remaining 2 mL was used for the preparation of serum. PBMCs were isolated for quantitative polymerase chain reaction (qPCR) using Ficoll-Hypaque (Lymphoprep; Nycomed Pharma, Oslo, Norway) density gradient centrifugation. Centrifugation was performed at 840 ×g for 20 min at 20°C. The serum was separated from the specimens and stored at −70°C until required for cytokine determination using an enzyme-linked immunosorbent assay (ELISA). All blood samples were obtained before the PE patients received treatments such as steroids or antihypertensive drugs.

### 2.4. RNA Isolation and RT-PCR

Total RNA was extracted from individual PBMC preparations using TRIzol reagent (Invitrogen, Carlsbad, CA, USA) according to the manufacturer's instructions. cDNA synthesis was performed using the High-Capacity cDNA Reverse Transcription kit (Applied Biosystems, Foster city, CA, USA) according to the manufacturer's instructions. Real-time PCR was performed in a 20 *μ*L system that contained 10 *μ*L of 1x SsoFast EvaGreen Supermix (Bio-Rad, Hercules, CA, USA), 2 *μ*L of cDNA, 6 *μ*L of RNase/DNase-free water, and 500 nM of each primer. The thermal cycler conditions were as follows: 30 s at 95°C, followed by 40 cycles of 5 s at 95°C and 10 s at 60°C. A melting curve analysis was performed for each reaction with a 65–95°C ramp. The threshold cycle at which the fluorescent signal reached an arbitrarily set threshold near the middle of the log-linear phase of the amplification for each reaction was calculated, and the relative quantity of mRNA was determined. The mRNA levels were normalized against the mRNA levels of the housekeeping gene, *β*-actin. The primer sequences for qPCR are shown in Table 2.

### 2.5. Cytokine Measurement Using ELISA

Serum IL-17A and IL-35 concentrations were measured by commercial ELISA according to the manufacturer's instructions (Bender MedSystems, Burlingame, CA, USA). The IL-17 family contains six members (IL-17A–F), with IL-17A being designated as the prototypic IL-17 cytokine. All samples were analyzed in duplicate. The samples were analyzed by the same staff in the same laboratory conditions. Within and between assay variations were less than 6% and 8% for all ELISA assays, respectively.

### 2.6. Statistical Analysis

Statistical analysis was performed with GraphPad Prism version 5.0 (GraphPad software Inc. San Diego, CA, USA). Data are presented as the means ± SD. *P* < 0.05 was considered to indicate a statistically significant difference. As determined by one-way analysis of variance (ANOVA) or Student's *t*-test. To test for associations, Pearson's correlation coefficients were calculated.

## 3. Results

### 3.1. The mRNA Expression of ROR*γ*t and Foxp3 in PBMC from PE Patients

**Figure 1:**
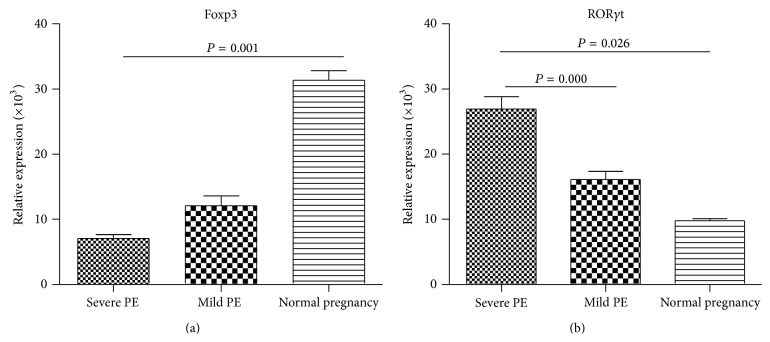
Expression of transcription factors in PBMC of PE patients and normal pregnant women. mRNA expression in PBMC from PE patients was measured by PCR. The result was normalized relative to beta actin and compared to expression of these mRNA from 22 normal pregnant women. (a) The relative expression of Foxp3 was compared among three groups; (b) the relative expression of ROR*γ*t was compared among three groups.

In this study, we determined the specific transcription factor of Th17 and Treg cells in three groups by real-time PCR. As shown in Figure 1, decreased mRNA expression of Treg specific transcription factor Foxp3 was observed in server PE patients compared with normal pregnant women (*P* < 0.001); as for Th17 specific transcription factor, ROR*γ*t mRNA expression was significantly higher in mild PE and severe PE patients than those normal controls (both *P* < 0.05).

### 3.2. The mRNA Expression of IL-17 and IL-35 Subunits (P35 and EBI3) in PE Patients

**Figure 2:**
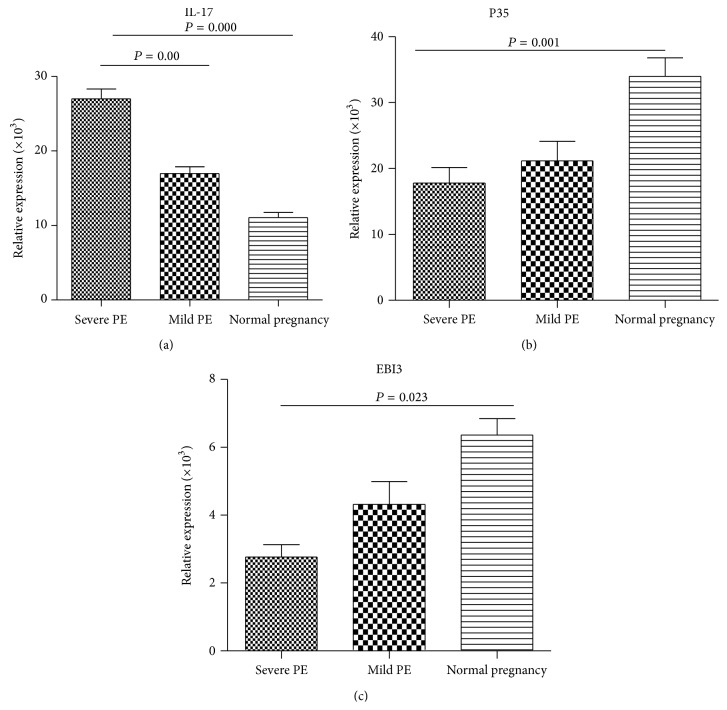
mRNA expression of IL-17 and IL-35 in PBMC from preeclampsia and normal pregnant women. (a) The mRNA expression of IL-17. (b) IL-35 subunit P35. (c) The expression of EBI3mRNA was decreased in PBMC from preeclampsia compared with normal pregnant women.

The results demonstrated that severe PE patients exhibited decreased mRNA expression levels of IL-35 subunits of P35 and EBI-3 (Figures 2(b) and 2(c)), whereas the IL-17 expression levels in mild and severe PE patients were higher than those normal pregnant women (Figure 2(a)).

### 3.3. Serum Cytokine Concentrations as Determined by ELISA

**Figure 3:**
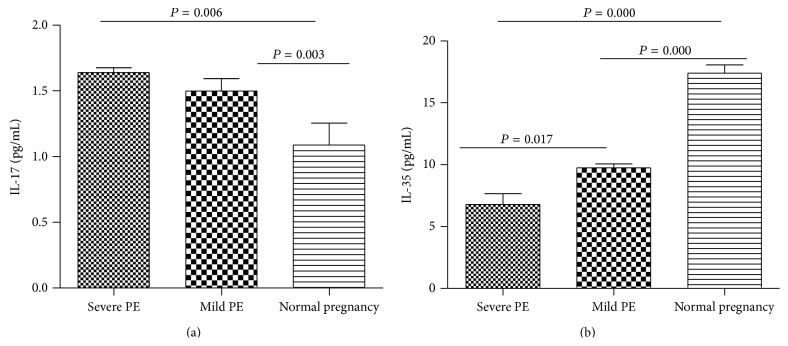
Serum cytokine concentrations were determined from the peripheral blood samples obtained from preeclampsia patients and normal pregnancy. (a) The concentrations of serum interleukin-17 (IL-17) and (b) interleukin-35 (IL-35) were examined by enzyme-linked immunosorbent assay (ELISA) using specific cytokine detection kits.

The expressions of Th17-related cytokines IL-17A and Treg-related cytokine IL-35 from PE patients were determined in the serum by ELISA as an indicator of cytokine production. As shown in Figure 3, the IL-17A expression levels in mild and severe PE patients were higher compared with the control group (Figure 3(a)). By contrast, the mild and severe PE patients demonstrated lower levels of IL-35 than normal pregnant women (Figure 3(b)). These data suggest an abnormal immune response in PE patients, characteristic of a shift to Th17-type immunity.

### 3.4. Expression of Notch/Notch Ligand in Preeclampsia Patients

**Figure 4:**
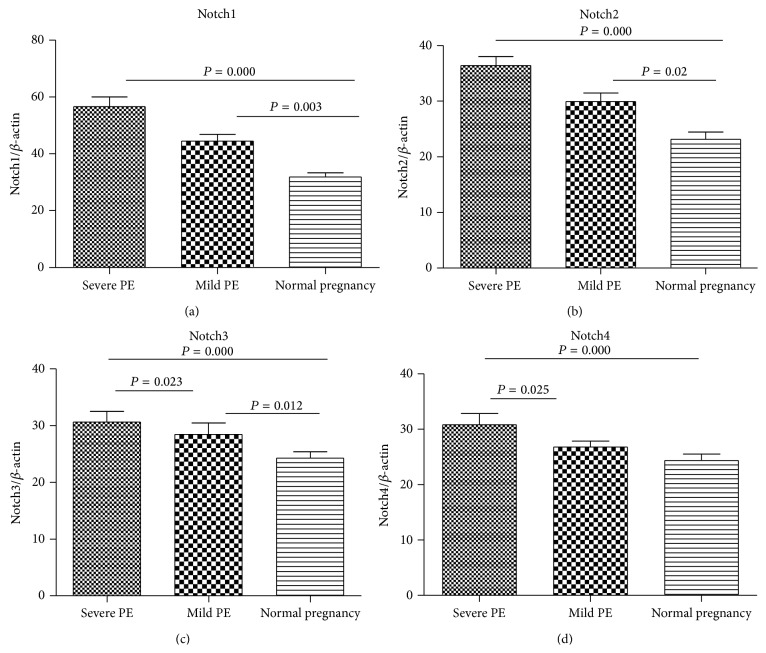
mRNA expression of Notch receptor in PBMC from preeclampsia and normal pregnant women. (a) The mRNA expression of Notch1; (b) the mRNA expression of Notch2; (c) the mRNA expression of Notch3; (d) the mRNA expression of Notch4. The data were normalized to a housekeeping gene beta actin.

Compared with normal pregnant women, monocytes from PE patients expressed higher level of Notch receptor (Figure 4) which suggested the high level Notch receptor may play an important role in the mechanism of PE.

**Figure 5:**
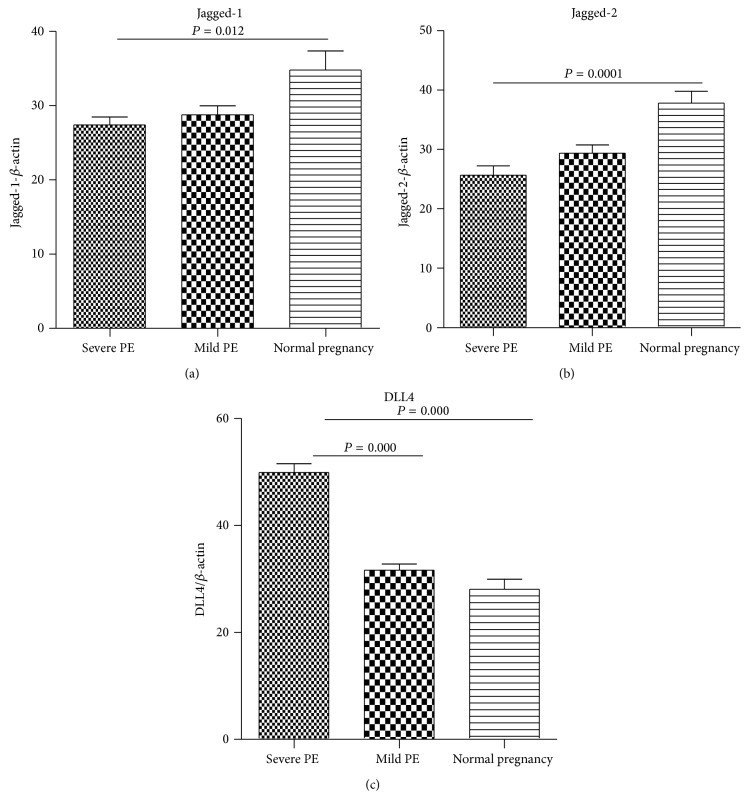
mRNA expression of Notch ligand in PBMC from preeclampsia and normal pregnant women. (a) The mRNA expression of Jagged-1; (b) the mRNA expression of Jagged-2; (c) the mRNA expression of DLL4. The data were normalized to a housekeeping gene beta actin.

Quantitative real-time PCR revealed that the mRNA of the Notch ligands DLL4 were expressed at very high level in mild and severe PE patients, whereas those of Jagged-(1-2) were expressed at very low level in severe PE patients (Figure 5).

### 3.5. Correlation between Notch Ligand and Th17 and Treg in Preeclampsia Patients

**Figure 6:**
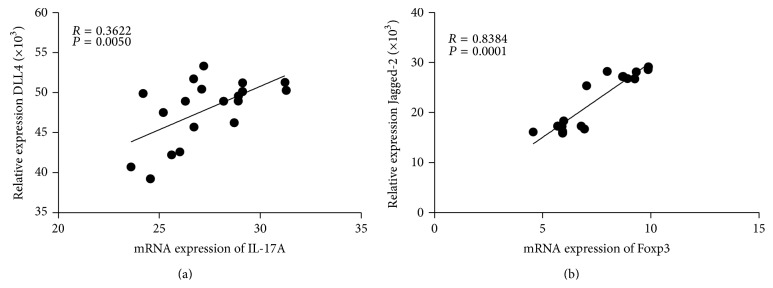
As shown in (a), there were positive correlations between the mRNA of IL-17 and DLL4 (*R* = 0.3622, *P* < 0.05), as well as between Foxp3 and Jagged-2 of Notch ligands (*R* = 0.8384; *P* < 0.001) in the patients of severe PE (b). It appears that Notch signaling is involved in the regulation of peripheral induced Th17 and Treg cells in PE patients.

As shown in Figure 6(a), there were positive correlations between the mRNA of IL-17 and DLL4 (*R* = 0.3622; *P* < 0.05), as well as between Foxp3 and Jagged-2 of Notch ligands (*R* = 0.8384; *P* < 0.001) in the patients of severe PE (Figure 6(b)). It appears that Notch signaling is involved in the regulation of peripheral induced Th17 and Treg cells in PE patients.

## 4. Discussion

In this study, we demonstrated a decrease in serum Treg-related cytokine IL-35, the mRNA expression of P35 and EBI-3 (two subunits of IL-35), and the Treg transcription factors Foxp3 in peripheral blood of PE patients. IL-35 is a recently discovered inhibitory cytokine that is secreted by Foxp3 regulator T cells for suppressive activity [19]. In the present study, we firstly detected the mRNA expression level of IL-35 subunits P35 and EBI3 in PBMC from PE patients. Our results show that the mRNA expression of P35 and EBI3 was both decreased obviously in PE patients. To further determine the expression and clinical significance of IL-35 with PE patients, we detected the expression level of IL-35 protein in the serum. The results were consistent with RT-PCR analysis of P35 and EBI3 (IL-35 subunits). Study results demonstrated that IL-35 is an important anti-inflammatory cytokines and can efficiently suppress the CD4^+^ effectors T cells (including Th1, Th2, and Th17) activity, induce the generation of Treg cells, and reduce the progression of inflammatory disease [20–23]. Our study found that PE patients exhibited a significantly decreased expression of Foxp3 and IL-35 in peripheral blood. One possible explanation is that low concentrations of IL-35 are due to the peripheral deficiency of Tregs, the major producer for this cytokine [24, 25]. Another explanation is that peripheral environment in PE state is not in favor of the differentiation of Treg cells.

Our study found that PE patients exhibited a significantly increased mRNA expression of transcription factor ROR*γ*t and IL-17 when compared with normal pregnancy. We next investigated the expression level of IL-17 protein in the serum of PE patients and normal pregnant women. We observed significantly increased IL-17 in serum from PE patients. Our study demonstrated that the anti-inflammatory cytokines IL-35 levels were significantly decreased in patients with PE, whereas the proinflammatory cytokines IL-17 levels were significantly increased in patients with PE. These results suggest a potential predominant role of Th17 cells in the PE patients [26]. Our study which is based on the analysis of Th17 and Treg production shows a dominance of Th17 cell activity over Treg cell activity that is observed in PE patients. Collectively, cytokine profile in peripheral blood of PE patients may provide suitable environment for the differentiation of Th17 cells, but not suitable for Treg cells. Thus, preeclampsia is characterized as a state of the excessive maternal inflammatory response with a predominance of the production of Th17 cells, suggesting systemic inflammation is a dominant component in the pathogenesis of preeclampsia [14, 27]. These results not only help to understand the etiology of preeclampsia, but also provide a novel rationale for the prevention or intervention of PE via regulation of the balance of Th17/Treg cells. Our results are consistent with recent data by Santner-Nanan et al. demonstrating that it is the balance between Tregs and Th17 cells that is critical to maintain tolerance to the fetus and prevents PE patients [28–30].

In the present study, we observed a marked increase of Notch1-4, and DLL4 with PE patients, but a decreased expression level of Jagged-1-2 levels with PE patients. In addition, we found that higher IL-17 levels were positively correlated with DLL4 in PE patients, whereas lower FoxP3 levels were positively correlated with Jagged-2 in severe PE patients, suggesting that the Treg and Th17 level not only is a potential predictor for symptoms onset of PE, but also is influenced by Notch signaling in PE patients. Several reports have shown that the presence of Notch/Notch ligands, mostly of the Jagged family, can enhance Treg cell differentiation and function in vitro, while DLL1 and DLL4 have been implicated in the development of Th1 or Th17 cells [31]. These data suggest that Notch-mediated signaling emerges as a key regulator of the development of T helper cell subsets promoting systemic inflammation, as well as other T helper cell subsets playing an anti-inflammatory role. Increasing evidence also indicates the involvement of Notch-mediated response in the pathogenesis of pregnancy [32–34]. Our studies suggest a role for Notch/Notch ligands in maintaining balance of Th17/Tregs cells during pathogenesis of PE patients.

## 5. Conclusion

Taken together, the homeostasis between Th17 and Treg cells might be essential for the fetus to be tolerated within the maternal environment. The imbalance of anti-inflammatory/proinflammatory cytokines plays an important role in the process of preeclampsia. The mechanism underlying the shift in cytokine profiles in preeclampsia remains undefined, but as the changes described here are likely to have evolved in vivo over time, they reflect the influence of a number of factors, including Notch signaling, Th17/Treg cells, IL-17, and IL-35 cytokines in the periphery of PE. Our findings of a positive correlation between the increased IL-17 and the upregulated DLL4 expression as well as between the decreased FoxP3 and the downregulated Jagged-2 expression may facilitate the potential development of novel therapeutic targeting for the treatment of the PE patients. 

## Figures and Tables

**Table 1:** The clinical characteristics of patients with preeclampsia and normal pregnancy.

	Mild PE	Severe PE	Normal pregnancy
	*N* = 22	*N* = 20	*N* = 22
Maternal age (years)	25.68 ± 2.50	26.37 ± 4.10	27.38 ± 3.38
Gestational age (weeks)	36.42 ± 1.62	36.62 ± 1.57	38.57 ± 0.98
Systolic pressure (mmHg)	147.27 ± 6.45^∗#^	167.25 ± 8.07^∗^	110.25 ± 8.07
Diastolic pressure (mmHg)	92 ± 3.74^∗^	116.33 ± 8.15^∗^	75.11 ± 5.58
MAP	115.33^∗^	146.67^∗^	82.67
Proteinuria (mg/24)	227.89 ± 28.61^#^	606.86 ± 74.45	Absent
Nulliparous	20 (90.9%)	18 (90.0%)	10 (45.5%)
Smokers	1 (4.6%)	1 (5.0%)	0 (0.0%)

MAP: mean arterial pressure = (2 × diastolic blood pressure + systolic blood pressure)/3; ^∗^ *P* < 0.05 mild and severe PE versus normal pregnancy; ^#^ *P* < 0.05 mild PE versus severe PE.

**Table 2:** Primers for RT-PCR.

Gene	Forward primer	Reverse primer
FoxP3	5′-TGAGAAGGACAGGGAGCCAA-3′	5′-GAGAAGCTGAGTGCCATGCA-3′
ROR*γ*t	5′-TGAGAAGGACAGGGAGCCAA-3′	5′-GAGAAGCTGAGTGCCATGCA-3′
IL-17	5′-CTCCAGAAGGCCCTCAGACTAC-3′	5′-GGGTCTTCATTGCGGTGG-3′
P35	5′-TCCTCCCTTGAAGAACCGGA-3′	5′-TGACAACGGTTTGGAGGGAC-3′
EBI3	5′-TCCTTCATTGCCACGTACAG-3′	5′-GCTCTGTTATGAAAGGCACG-3′
Notch1	5′-TGCCAAGCTCAGTGGTGTTGTA-3′	5′-TGCTAGGCTTTGTGGGATTCAG-3′
Notch2	5′-GGTTCCCAGTGAGCACCCTTAC-3′	5′-GTGGATTCGGACCAGTCTGAGAG-3′
Notch3	5′-ACCTGCTCAACGGCTTCCA-3′	5′-AGCTTCTGCACTCATCGATATCCTC-3′
Notch4	5′-ACCTGCTCAACGGCTTCCA-3′	5′-AGCTTCTGCACTCATCGATATCCTC-3′
Jagged-1	5′-ACCAGGTGGACGGCTTTGAG-3′	5′-CCCGGGATGCAATCACAGTAATA-3′
Jagged-2	5′-TGGGCTACTCCGGCTTCAAC-3′	5′-ACAGGTAGGCATCACCGAGGTC-3′
DL-4	5′-GACTGTGAAGCACCTCCG-3′	5′-GAAGTCCCTCACCCTCCCAA-3′
*β*-actin	5′-TGGCACCCAGCACAATGAA-3′	5′-CTAAGTCATAGTCCGCCTAGAAGCA-3′

## Abbreviations

PE: Preeclampsia
PBMCs: Peripheral blood mononuclear cells
Th: T helper
IL-17: Interleukin-17
IL-35: Interleukin-35
EBI3: Epstein-Barr-virus- (EBV-) induced gene 3
Treg cells: Regulatory T cells
ROR*γ*t: Orphan receptor-*γ*-t
FoxP3: Forkhead Box protein 3
DLL4: Delta-like 4
BP: Blood pressure
MAP: Mean arterial pressure
ELISA: Enzyme-linked immunosorbent assay.

## References

[1] Le J. (2008). *Obstetrics and Gynecology*.

[2] Cunningham F. G., Williams J. W. (2010). *Williams Obstetrics*.

[3] Comabella M., Khoury S. J. (2012). Immunopathogenesis of multiple sclerosis. *Clinical Immunology*.

[4] Saito S., Nakashima A., Shima T., Ito M. (2010). Th1/Th2/Th17 and Regulatory T-cell paradigm in pregnancy. *The American Journal of Reproductive Immunology*.

[5] Cao W., Xu W., Chen T. (2014). CD4^+^CD25^+^FoxP3^+^ regulatory T cells and cytokines interact with estradiol in cases of missed abortion. *Experimental and Therapeutic Medicine*.

[6] Erlebacher A. (2013). Mechanisms of T cell tolerance towards the allogeneic fetus. *Nature Reviews Immunology*.

[7] Samstein R. M., Josefowicz S. Z., Arvey A., Treuting P. M., Rudensky A. Y. (2012). Extrathymic generation of regulatory T cells in placental mammals mitigates maternal-fetal conflict. *Cell*.

[8] Collison L. W., Workman C. J., Kuo T. T. (2007). The inhibitory cytokine IL-35 contributes to regulatory T-cell function. *Nature*.

[9] Ozkan Z. S., Simsek M., Ilhan F., Deveci D., Godekmerdan A., Sapmaz E. (2014). Plasma IL-17, IL-35, interferon-*γ*, SOCS3 and TGF-*β* levels in pregnant women with preeclampsia, and their relation with severity of disease. *The Journal of Maternal-Fetal & Neonatal Medicine*.

[10] Whitehead G. S., Wilson R. H., Nakano K., Burch L. H., Nakano H., Cook D. N. (2012). IL-35 production by inducible costimulator (ICOS)-positive regulatory T cells reverses established IL-17-dependent allergic airways disease. *Journal of Allergy and Clinical Immunology*.

[11] Crome S. Q., Wang A. Y., Levings M. K. (2010). Translational mini-review series on Th17 cells: function and regulation of human T helper 17 cells in health and disease. *Clinical and Experimental Immunology*.

[12] Banchereau J., Pascual V., O'Garra A. (2012). From IL-2 to IL-37: the expanding spectrum of anti-inflammatory cytokines. *Nature Immunology*.

[13] Laresgoiti-Servitje E. (2013). A leading role for the immune system in the pathophysiology of preeclampsia. *Journal of Leukocyte Biology*.

[14] Saito S. (2010). Th17 cells and regulatory T cells: new light on pathophysiology of preeclampsia. *Immunology and Cell Biology*.

[15] Darmochwal-Kolarz D., Kludka-Sternik M., Tabarkiewicz J. (2012). The predominance of Th17 lymphocytes and decreased number and function of Treg cells in preeclampsia. *Journal of Reproductive Immunology*.

[16] Radtke F., Fasnacht N., MacDonald H. R. (2010). Notch signaling in the immune system. *Immunity*.

[17] Taghon T., Waegemans E., Van de Walle I. (2012). Notch signaling during human T cell development. *Microbiology and Immunology*.

[18] Radtke F., MacDonald H. R., Tacchini-Cottier F. (2013). Regulation of innate and adaptive immunity by Notch. *Nature Reviews Immunology*.

[19] Chaturvedi V., Collison L. W., Guy C. S., Workman C. J., Vignali D. A. A. (2011). Cutting edge: human regulatory T cells require IL-35 to mediate suppression and infectious tolerance. *Journal of Immunology*.

[20] Collison L. W., Chaturvedi V., Henderson A. L. (2010). IL-35-mediated induction of a potent regulatory T cell population. *Nature Immunology*.

[21] Prins J. R., Boelens H. M., Heimweg J. (2009). Preeclampsia is associated with lower percentages of regulatory T cells in maternal blood. *Hypertension in Pregnancy*.

[22] Kassan M., Wecker A., Kadowitz P., Trebak M., Matrougui K. (2013). CD4^+^CD25^+^Foxp3 regulatory T cells and vascular dysfunction in hypertension. *Journal of Hypertension*.

[23] Kassan M., Wecker A., Kadowitz P., Trebak M., Matrougui K. (2013). CD4^+^CD25+Foxp3 regulatory T cells and vascular dysfunction in hypertension. *Journal of Hypertension*.

[24] Hanidziar D., Koulmanda M. (2010). Inflammation and the balance of Treg and Th17 cells in transplant rejection and tolerance. *Current Opinion in Organ Transplantation*.

[25] Littman D. R., Rudensky A. Y. (2010). Th17 and regulatory T cells in mediating and restraining inflammation. *Cell*.

[26] Sasaki Y., Darmochwal-Kolarz D., Suzuki D. (2007). Proportion of peripheral blood and decidual CD4^+^CD25^bright^ regulatory T cells in pre-eclampsia. *Clinical and Experimental Immunology*.

[27] Gratz I. K., Rosenblum M. D., Maurano M. M. (2014). Cutting edge: self-antigen controls the balance between effector and regulatory T cells in peripheral tissues. *The Journal of Immunology*.

[28] Toldi G., Molvarec A., Stenczer B. (2011). Peripheral T_h_1/T_h_2/T_h_17/regulatory T-cell balance in asthmatic pregnancy. *International Immunology*.

[29] Steinborn A., Haensch G. M., Mahnke K. (2008). Distinct subsets of regulatory T cells during pregnancy: is the imbalance of these subsets involved in the pathogenesis of preeclampsia?. *Clinical Immunology*.

[30] Santner-Nanan B., Peek M. J., Khanam R. (2009). Systemic increase in the ratio between Foxp3^+^ and IL-17-producing CD4^+^ T cells in healthy pregnancy but not in preeclampsia. *The Journal of Immunology*.

[31] Carlin S. M., Khoo M. L. M., Ma D. D., Moore J. J. (2012). Notch signaling inhibits CD4 expression during initiation and differentiation of human T cell lineage. *PLoS ONE*.

[32] Auderset F., Coutaz M., Tacchini-Cottier F. (2012). The role of notch in the differentiation of CD4^+^ T helper cells. *Current Topics in Microbiology and Immunology*.

[33] Amsen D., Antov A., Flavell R. A. (2009). The different faces of Notch in T-helper-cell differentiation. *Nature Reviews Immunology*.

[34] Artavanis-Tsakonas S., Muskavitch M. A. T. (2010). Notch: the past, the present, and the future. *Current Topics in Developmental Biology*.

